# Trofinetide receives FDA approval as first drug for Rett syndrome

**DOI:** 10.1097/MS9.0000000000001896

**Published:** 2024-03-18

**Authors:** Zaib Un Nisa Mughal, Bisma Ahmed, Burhanuddin Sohail Rangwala, Hussain Sohail Rangwala, Hareer Fatima, Mirha Ali, Asma Ahmed Farah

**Affiliations:** a Department of Medicine, Jinnah Sindh Medical University, Karachi, Sindh, Pakistan; b Department of Medicine, East Africa University, Boosaaso, Somalia

## Introduction

Rett syndrome (RTT) is a neurodevelopmental illness affecting around 1 in every 10,000 live births, almost exclusively affecting females^1^. RTT’s key traits are aberrant linguistic and psychomotor growth (it refers to abnormal or deviating patterns of development in language skills and motor coordination, this deviation may manifest as atypical linguistic abilities and challenges in developing motor skills), autistic behaviors, erratic breathing, uneven walking, and hand wringing^2^. The early postnatal period saw regular neurological and physical growth, which leads to symptom emergence between 6 and 18 months of age^3^. The four phases of the RTT progression (Table 1)^3^ are Stage 1 (early onset), Stage 2 (regression), Stage 3 (plateau), and Stage 4 (late motor deterioration). In Stage 1 (early onset), children grow normally, albeit there may be some developmental delay. Stage 2, known as regression, sees youngsters regress and lose previously learned abilities, such as deliberate hand gestures and spoken speech. Children’s cognitive skills stabilize at Stage 3 (plateau) when other medical issues start to manifest themselves like seizures. People with RTT endure declining mobility in Stage 4 (late motor degeneration), and they may start to exhibit parkinsonian symptoms^4^.

**Table 1:** Rett syndrome evolves via various phases^3^

Stage	Age	Symptoms
1. Stagnation	6–18 months	Developmental delays (postural control, motor, language) Reduced eye contact Hand-wringing may occur Microcephaly may occur
2. Rapid regression	1–4 years	Loss of purposeful hand skills Stereotypical hand movements (wringing, washing, tapping) Loss of spoken language Walking may be unsteady Breathing irregularities may occur Autistic-like features Microcephaly progression Seizures may occur
3. Plateau/pseudo-stationary	2–potentially life	Hand apraxia/dyspraxia Motor coordination difficulties and/or loss of motor skills Improvement of communication skills may occur Seizures are common
4. Late motor deterioration	10–life	Severe physical disability Muscle weakness, rigidity, or spasticity Wheelchair dependency may occur

The RTT brain has a diminished volume comparable to that of an ideal 1-year-old kid. RTT is associated with a reduction in brain volume, particularly affecting regions such as the frontal and temporal lobes. The diminished volume observed in individuals with RTT is often characterized by a loss of neurons and a decrease in dendritic branching. Specific brain areas like the cerebral cortex, which plays a crucial role in various cognitive functions, may exhibit reduced size and altered structure. This abnormality contributes to the neurological and developmental challenges associated with RTT. It is stable for at least 40 years without exhibiting any signs of neurodegeneration. Patients with RTT exhibit abnormal movements during pregnancy and the first year of life, which can result in microcephaly. It is thought that the primary reasons are imbalances in dendritic arborization, synaptic density, neuronal soma size, and increased cell density^5^. Several patients have impaired brain carbohydrate metabolism, dyslipidemia, raised plasma leptin and adiponectin, abnormal mitochondrial structure, raised oxidative stress, lactate and pyruvate levels, GI issues, altered QT intervals, diminished bone mass, osteoporosis, gallbladder inflammation, scoliosis, urological dysfunction, and sleep disruptions. The fact that several tissues and systems are involved emphasizes the fact that RTT is a multi-systemic illness^6^.

## Pathogenesis of Rett syndrome

An X chromosome-linked gene called Methyl-CpG-binding protein 2 (MeCP2) produces the MeCP2 protein, which plays an essential role in the RTT^7^. MeCP2 is highly produced in the nucleus and is essential for the differentiation, maturity, improvement of neural circuits, and maintenance of synapses. Early in the embryonic process, it is low. MeCP2 has two primary isoforms, MeCP2-E1 and MeCP2-E2, distinct by amino (N)-terminal amino-acid residues^8^. MeCP2 has two conserved domains: transcriptional repression and methyl-DNA binding^7^. Because of its affinity for methylated CpG sites linked to transcriptional silence, MeCP2 was first recognized as a repressor^9^. However, when it binds to 5-hydroxymethylcytosine, a frequent alteration of DNA in the brain that is concentrated in active genes, it has also been proposed as a transcription activator^10^. In RTT, C > T type nucleotide transition mutations are found in CpG hotspots and most likely represent variable site methylation in the male germline. Only female progeny can be affected by RTT mutations, which develop spontaneously in the paternal germline cells^11^.

More than 200 distinct MeCP2 mutations have been linked to RTT. R106W, R133C, T158M, R168X, R255X, R270X, R294X, and R306C are the most prevalent point mutations, accounting for nearly 60% of RTT instances^4^. Microarray research revealed that WNT6 mRNA levels were 12-fold reduced in the CA1 zone of the hippocampus of MeCP2K412R sumo mutant mice (MeCP2 Lys-412 residue sumo-mutant animals)^12^. Wnt proteins and the Wnt/-catenin pathway are crucial for proper brain development. It has also been emphasized as a hotspot for autism signaling. Wnt6 signaling dysfunction and its downstream effectors may be involved in the etiology of RTT, as evidenced by decreased MeCP2 SUMOylation and reduced Wnt6 mRNA levels in MeCP2K412R sumo-mutant animals^7^.

## Treatment of Rett syndrome

### Old treatments

Due to its multi-systemic dysfunction, RTT requires a multifaceted strategy for medical therapy and management^13^. Patients die as a result of heart failure, pulmonary infection, or respiratory collapse^6^. The majority of treatment techniques aim to stimulate MeCP2 gene expression and control its downstream mechanisms. Actinomycin-D suppresses MeCP2 repression, Tamoxifen restores MeCP2 expression, IGF-1 tripeptide improves life expectancy in MeCP2 mutant mice, and Valproic acid is an HDAC blocker that serves to treat seizure disorder^14^. Ketamine administration bettered limb clasping, muscle control, and breathlessness in MeCP2-mutant mice. Desipramine, a norepinephrine reuptake inhibitor, alleviated MeCP2-mutant mice’s ventilation abnormalities and apneas. Sarizotan, a serotonin 1a agonist and Dopamine D2-like receptor agonist, lowered breathlessness by 15–30% but had no influence on motor function in MeCP2-mutant mice^3^. Fluoxetine and buspirone had positive effects on respiratory dysfunction in RTT cases. Levodopa and benserazide administration alone refined RTT traits, dendrite growth, spine density, and lifespan. Choline therapy bettered motor efficiency, grasping power, nerve cell growth and behavioral impairments in RTT mouse models^14^. Vitamin E derivative Trolox alleviates neural cells hyperactivity while also increasing synaptic remodeling and hypoxia tolerance. N-acetylcysteine modulates oxidative stress by increasing intracellular glutathione levels in MeCP2/y mice^15^. Fluvastatin and Lovastatin restore cholesterol metabolism, Phenytoin prevents arrhythmias, Zoledronic acid treats osteoporosis and lowers the incidence of fractures, Folate and Betaine recover RTT morphology by promoting DNA methylation of CpG binding proteins. Dextromethorphan is an NMDA receptor blocker entirely for mental functioning and convulsions, while Topiramate TPM therapy improved seizure control and respiratory function in RTT patients. Levetiracetam is a powerful anticonvulsant medication for RTT patients, whereas Naltrexone is an opiate blocker used orally that boosts breathing efficiency but has little impact on the anticipated psychomotor handicap^13,14^.

## New treatments

### Trofinetide

Trofinetide, an oral medication sold under the brand name Daybue, has just received FDA approval for the treatment of RTT, an uncommon gene-related disorder that impairs neurological growth, in adults and children 2 years of age and older. Before 10 March 2023, there were no FDA-endorsed treatments available for RTT, with the primary focus on managing the syndrome’s symptoms and impacts through a multidisciplinary and interprofessional approach. Trofinetide became an inaugural FDA-approved treatment for RTT on 10 March 2023. Trofinetide (glycyl-L-2-methylprolyl-L-glutamic acid) is a brand-new synthetic analogue of the brain protein glycine-proline-glutamate (GPE). Vomiting and diarrhea are frequent side effects of the twice-daily oral medication, according to an FDA statement^16^. Diminution in weight, Fever, convulsions, anxiousness, reduced satiety, exhaustion and nasopharyngitis are some of the additional adverse effects. Trofinetide’s mechanism of action in RTT patients remains unclear. Trofinetide has been found in animal research to promote dendritic branching as well as neuronal plasticity signals^17^.

In a phase 2 placebo-controlled trial involving 82 female mice with RTT aged 5–15 years, trofinetide, exhibited promising results. In MeCP2-deficient mice, GPE improves neurological, pulmonary and cardiovascular performance, as well as heart rate, increases brain mass and lengthens survival. Both GPE and Trofinetide, which has a prolonged half-life than GPE, halted cell death and decreased infarct volume in a rat model of hypoxic insult, with Trofinetide showing a dose-dependent decrease in infarct volume^18^. The highest trofinetide dose (200 mg/kg twice daily [BID]) showed a significant improvement (*P* ≤ 0.042) over the placebo on three key measures: the RTT Behavior Questionnaire (RSBQ), Clinical Global Impression-Improvement (CGI-I), and RTT-Clinician Domain Specific Concerns-Visual Analog Scale (RTT-DSC-VAS). The mice tolerated trofinetide well at all doses tested (50, 100, and 200 mg/kg BID). Encouraged by these findings, a phase 3 trial is now underway, employing disease-specific and innovative scales to further investigate the efficacy and safety of trofinetide in female mice with RTT^18^.

Variations in Trofinetide bioavailability between morning and evening doses may have significant clinical implications. This information may guide the development of more personalized treatment plans, considering chronobiological factors and tailoring doses to align with the symptomatology patterns. Optimizing dosing schedules could potentially enhance the efficacy of trofinetide in managing RTT. However, ensuring patient adherence is crucial and requires education on the importance of consistent dosing. In addition, regular monitoring and potential adjustments in dosage timing based on individual responses may be necessary to maintain stable therapeutic effects. Overall, a deeper understanding of trofinetide bioavailability changes throughout the day could refine treatment strategies to achieve better outcomes in patients with RTT^19^.

In a phase 2 study, trofinetide was administered orally twice daily in three weight-based dosages (50 mg/kg, 100 mg/kg, or 200 mg/kg) for 6 weeks to 82 children and adolescents with RTT. The 200 mg/kg dose significantly improved efficacy measures as determined by care takers and medical professionals^20^. Oral trofinetide administration resulted in linear kinetics in early clinical pharmacokinetic studies (including population pharmacokinetic modeling in healthy adult participants), with no time- or dose-dependent effects on pharmacokinetic parameters. Trofinetide did not induce or inhibit any metabolic processes during the dose range that was the subject of the study (up to 200 mg/kg), and systemic exposure was dose-proportional throughout. Following administration of numerous doses, there was little to no buildup. Trofinetide’s bioavailability was found to change between morning and evening doses in these early tests, which may have been caused by a dietary effect or circadian oscillations in metabolism or absorption. In the population pharmacokinetic modeling study, a two-compartment model best represented the pharmacokinetic parameters of Trofinetide; nevertheless, noncompartmental analysis is often preferred for characterizing pharmacokinetics in a single study. Concurrent food consumption may alter the pharmacokinetic profile of orally delivered medications by a variety of methods, such as postponing stomach emptying, altering the gastrointestinal tract’s pH, or altering a drug’s luminal metabolism. The main goal of the current investigation was to identify any potential interactions between food and Trofinetide pharmacokinetic parameters after oral administration of patients with RTT at the highest recommended clinical dose (12 g). Trofinetide’s pharmacokinetic characteristics were compared between morning and evening administration in order to identify any potential diurnal fluctuation in bioavailability. The study’s second exploratory goal was to describe the pharmacokinetics of Trofinetide in urine. The selection of weight-based dosages in a phase 2 study is often grounded in determining the optimal balance between efficacy and safety across a diverse patient population. By tailoring dosages to different weight categories, researchers can assess how variations in body weight influence drug responses and potential side effects. This approach helps identify appropriate dosage ranges for further investigation, aiming to establish a more personalized and effective treatment strategy for a broad spectrum of individuals within the target population^21^.

## Conclusion

The approval of Trofinetide as the first drug for RTT is a significant breakthrough for families affected by this rare and debilitating condition. For many years, there has been no cure for RTT, and treatment options have been limited to managing the symptoms of the condition. Trofinetide offers hope that some of the cognitive, behavioral, and language impairments associated with RTT can be improved.

While Trofinetide is not a cure, its approval by the FDA represents a major milestone in the development of treatments for RTT. The drug has undergone rigorous clinical testing and has been shown to be safe and effective for improving some of the symptoms of the condition. It will be available in two formulations, offering flexibility and choice to patients and their families.

Looking ahead, it is hoped that further research will continue to refine and improve the effectiveness of Trofinetide and that additional treatments will be developed to address the underlying causes of RTT. However, for now, the approval of Trofinetide represents a significant step forward in the treatment of RTT and offers hope to the many families affected by this condition.

## Ethical approval

Ethics approval was not required for this editorial.

## Consent

Informed consent was not required for this editorial.

## Sources of funding

The authors received no extramural funding for the study.

## Author contribution

The conceptualization was done by Z.U.N.M. and B.S.R; literature and drafting of the manuscript were conducted by Z.U.N.M., B.A., F.A., A.S., B.S.R., H.S.R., M.A., and A.A.F.; editing and supervision were performed by B.S.R. All authors have read and agreed to the final version of the manuscript.

## Conflicts of interest disclosure

The authors declare no potential conflicts of interest concerning the research, authorship, and/or publication of this article.

## Research registration unique identifying number (UIN)

Not applicable.

## Guarantor

Not applicable.

## Data availability statement

Not applicable.

## Provenance and peer review

Not applicable.
